# Iatrogenic Hypercalcaemia Secondary to Antibiotic-Eluting Absorbable Calcium-Sulphate Beads in Orthopaedic Surgery

**DOI:** 10.7759/cureus.91009

**Published:** 2025-08-26

**Authors:** Arit C Akiba, Ola Yousif, Douglas Bairstow

**Affiliations:** 1 Trauma and Orthopaedics, South Tees NHS Foundation Trust, Middlesbrough, GBR

**Keywords:** antibiotic eluting implants, calcium sulfate beads, girdlestone resection arthroplasty, iatrogenic hypercalcemia, orthopaedic surgery, periprosthetic joint infection

## Abstract

Calcium-sulphate beads (CSBs) are increasingly used to manage surgical site infections, serving both as local antibiotic carriers and as fillers for dead space. Although generally well tolerated, hypercalcaemia has been described as a serious adverse effect. In this case report, we describe a patient who developed symptomatic hypercalcaemia secondary to using CSBs during a proximal femoral excision (Girdlestone procedure).

This study describes an 86-year-old woman who developed decreased consciousness and altered neurological status four days post a Girdlestone procedure with placement of 20 cc of antibiotic-eluting CSBs. She was afebrile with normal cardiovascular, respiratory, and abdominal examinations but exhibited abnormal neurological findings, responding only to painful stimuli. Laboratory results revealed severe hypercalcaemia. She had no past medical history of malignancy, parathyroid, or calcium disorders. She was subsequently treated with IV fluids and an IV zoledronic acid infusion. Over the following week, her clinical status improved and hypercalcaemia resolved.

This case highlights that significant hypercalcaemia can still occur outside the traditionally recognised risk factors, emphasising the need for vigilance even in lower-risk patients.

## Introduction

Prosthetic joint infections (PJIs) have become a serious and growing issue in orthopaedic surgery, with an incidence of 0.6-2.2% after primary total hip arthroplasty [1]. Surgery is widely regarded as the gold standard in managing PJIs, often requiring multi-modal approaches [2]. It typically involves repeated debridement of affected tissues, irrigation with antibiotic-containing solutions, and insertion of antibiotic-eluting beads [2].

Calcium-sulphate beads (CSBs) are bio-absorbable antibiotic carriers that can be used to manage surgical site infections as well as fill dead space [3]. They have become increasingly used in managing PJIs due to their ability to deliver and sustain high local antibiotic concentrations at the infection site over several weeks. In our case, Stimulan® (Biocomposite, UK), a commercially available CSBs was used. It has been shown to deliver 100% of its antibiotic load at the affected site over several weeks [4]. Other local antibiotic carriers have previously been used, such as polymethylmethacrylate (PMMA), but the advantage of CSBs is they are fully resorbable, eliminating the need for a second-stage removal surgery, unlike the alternative [5].

While generally considered to be safe, rare adverse effects have been reported with the use of CSBs, including cases of hypercalcaemia, typically associated with higher blood volumes or associated renal impairment [6-9]. Hypercalcaemia can be life-threatening, leading to neurological symptoms, cardiac arrhythmias, renal dysfunction, and in severe cases, coma if not recognised promptly.

We present a case of an elderly woman who presented symptomatic hypercalcaemia four days after placement of CSBs for a right prosthetic hip infection. This case highlights the need for clinician awareness of this rare complication, even in patients without traditional risk factors, and underscores the importance of postoperative vigilance.

## Case presentation

An 86-year-old Caucasian woman presented with altered neurological status four days after undergoing an excision arthroplasty (Girdlestone procedure) on her right hip with insertion of antibiotic-eluting CSBs. Her medical history included osteoporosis, obesity, iron deficiency anaemia, age-related macular degeneration, and a previous left intertrochanteric hip fracture managed with a dynamic hip screw.

Her regular medications were alendronic acid 70 mg weekly, citalopram 50 mg daily, famotidine 20 mg daily, propranolol 80 mg daily, and cholecalciferol 400 units/calcium carbonate 1.5 g twice daily, providing a total daily elemental calcium intake of approximately 1,200 mg.

This patient initially presented with a right hip fracture managed with a hip hemiarthroplasty. She was transferred to a district hospital for rehabilitation five days postoperatively. 19 days later, she was readmitted with a right periprosthetic hip joint infection and underwent the DAIR (debridement, antibiotic, and implant retention) procedure. The DAIR involved surgical debridement of infected tissue, targeted antibiotic therapy, and retention of the existing prosthesis.

**Figure 1:**
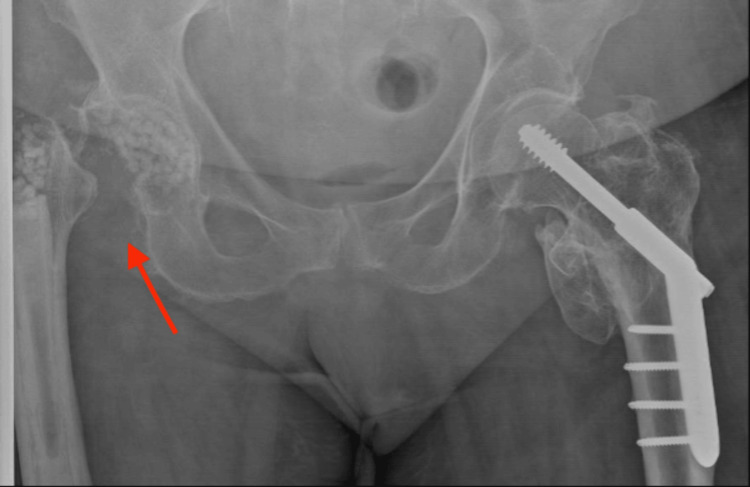
AP pelvis X-ray. The red arrow points to the right proximal femoral excision (Girdlestone procedure) with placement of antibiotic-eluting CSBs. AP: Anteroposterior; CSB: Calcium-sulphate bead

The infection persisted despite the DAIR procedure, so two weeks later, she underwent a Girdlestone procedure involving he removal of her femoral head and neck, with insertion of 20 cc of antibiotic-eluting CSB loaded with gentamicin and vancomycin as shown in Figure 1. 

On postoperative day four, she developed decreased consciousness, responding only to painful stimuli, with a Glasgow Coma Scale (GCS) score of 8 (E2V2M4). Preoperatively, her corrected serum calcium was 2.57 mmol/L (reference range: 2.2-2.6 mmol/L). No routine preoperative parathyroid hormone (PTH) measurement was undertaken, but her most recent PTH, taken two weeks earlier, was 5.4 pmol/L (reference range: 1.3-7.3 pmol/L), which was within the normal range. At presentation, laboratory results revealed an elevated corrected serum calcium of 3.34 mmol/L. Her estimated glomerular filtration rate (eGFR) was 68 ml/min (>90 ml/min), consistent with her baseline, and 25OH-cholecalciferol (Vitamin D) was 47.0 nmol/L (reference range: <30 nmol/L = deficient, 30-50 nmol/L = insufficient, >50 nmol/L = adequate) (as detailed in Table 1).

**Table 1:** Laboratory results on postoperative day 3

Variable	Value	Reference range
Total corrected serum calcium (mmol/L)	3.34	2.2-2.6
Creatinine (µmol/L)	131	50-100
25OH-cholecalciferol (Vitamin D) (nmol/L)	47	<30: Deficient; 30-50: Insufficient; >50: Adequate

**Figure 2:**
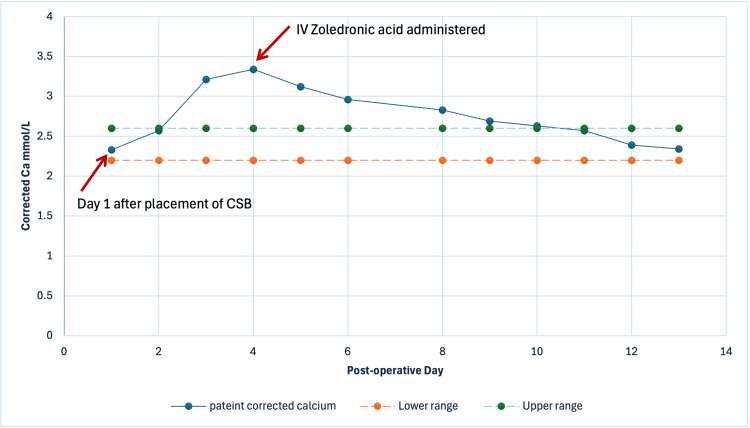
Graph showing calcium levels during hospitalisation

She was treated with IV fluids and a single dose of IV bisphosphonates (4 mg of zoledronic acid). Within 48 hours of treatment initiation, her neurological status improved with GCS score of 15. This improvement correlated with the downward trend of her serum calcium levels. No further bisphosphonate therapy was required, and her calcium level normalised within seven days of treatment initiation as outlined in Figure 2. No other cause of acute hypercalcaemia was discovered during her hospital admission.

Given the absence of any other identifiable cause of her hypercalcaemia, the timing of the symptom onset, and the rapid postoperative progression, the elevated serum calcium level was attributed to the antibiotic-eluting CSB, exacerbated by the patient’s prolonged immobilisation from hospitalisation and critical illness related to infection.

## Discussion

This case highlights symptomatic hypercalcaemia as an often under-recognised complication of CSBs use. While CSBs are increasingly used as local antibiotic carriers in the treatment of PJIs, they are not without potential systemic effects [4]. Hypercalcaemia has been reported in a small number of patients, typically those receiving high volumes of beads or those with underlying renal impairment. However, our patient developed severe, symptomatic hypercalcaemia despite having preserved renal function and receiving only a moderate volume of CSBs, suggesting that even lower doses can pose risks in certain individuals.

The largest reported case series involving CSBs included 755 patients treated with antibiotic-eluting Stimulan® beads [6]. In this study, 41 patients (5.4%) developed transient hypercalcaemia, with mean calcium levels of 11.7 mg/dL (range: 10.8-14.9 mg/dL). Only two patients became symptomatic, emphasising the relative rarity of clinically significant cases [6]. Importantly, the study found a positive correlation between CSB volume and risk of hypercalcaemia, suggesting a dose-dependent relationship. Based on these findings, the authors recommended limiting CSB volume to no more than 40 cc per procedure, or up to 80 cc when placed within the medullary canal [6].

The proposed mechanism of the hypercalcaemia involves gradual CSBs resorption and the subsequent release of calcium ions into surrounding tissues, which are then absorbed into the systemic circulation via rich capillary networks [7]. While many reported cases involved larger bead volumes, symptomatic hypercalcaemia has also been observed with volumes as low as 20 cc, as seen in our patient [8].

Hypercalcaemia typically presents within three to seven days postoperatively, with neurological symptoms ranging from lethargy and confusion to reduced consciousness and coma [7]. These non-specific features can easily be misattributed to other postoperative complications, particularly in elderly or frail patients. Hence, awareness and a high index of suspicion are essential.

A thorough diagnostic workup is critical to exclude alternative causes such as malignancy, primary hyperparathyroidism, and vitamin D toxicity [8]. In our case, unfortunately, no PTH level was obtained at the time of the hypercalcaemic episode due to the patient’s acute clinical deterioration, where the priority was stabilisation and urgent management. However, a PTH measurement performed two weeks earlier was within the normal range, and vitamin D levels were insufficient but not excessive, making CSBs the most likely source of calcium overload.

Management of CSB-related hypercalcaemia follows standard treatment protocols, including IV fluid resuscitation and bisphosphonates such as zoledronic acid [9]. In more severe cases, particularly those with renal impairment or resistant hypercalcaemia, haemodialysis may be required [9]. Our patient responded well to supportive care and a single dose of IV zoledronic acid, with normalisation of calcium levels within a week, consistent with other published cases [6].

## Conclusions

This case highlights that symptomatic hypercalcaemia, although rare, can occur even with moderate volumes of CSBs and in the absence of traditionally recognised risk factors such as renal impairment. Clinicians should therefore maintain a high index of suspicion for this complication in postoperative patients presenting with altered mental status, particularly in the elderly. In any patient presenting with neurological symptoms post-CSB implantation, serum calcium should be urgently evaluated.

In this patient, vulnerability factors such as advanced age, recent systemic inflammation related to PJI, and critical illness requiring surgery may have increased the risk of developing hypercalcaemia, even at a lower CSB volume.

Notably, there is currently no consensus on postoperative monitoring protocols for patients receiving CSBs. Based on this case and available literature, we suggest that in higher-risk groups including older adults, those with systemic infection or prolonged immobility, serum calcium should be monitored around days 3 and 7 postoperatively, which correspond to the typical window for symptom onset.

Further research is needed to establish formal guidelines, identify high-risk patient populations more precisely, and define safe CSB dosing thresholds to minimise this potentially serious complication. This case exemplifies the importance of such guidance, especially in older adults with acute infections.
